# Synthesis of a Novel Zn-Salphen Building Block and Its Acrylic Terpolymer Counterparts as Tunable Supramolecular Recognition Systems

**DOI:** 10.3390/molecules24122245

**Published:** 2019-06-15

**Authors:** Gustavo A. Zelada-Guillén, Ana B. Cuéllar-Sánchez, Margarita Romero-Ávila, Martha V. Escárcega-Bobadilla

**Affiliations:** School of Chemistry, National Autonomous University of Mexico (UNAM), Circuito Escolar s/n, Ciudad Universitaria, Mexico City 04510, Mexico; anacue506@gmail.com (A.B.C.-S.); mago_ro@comunidad.unam.mx (M.R.-Á.)

**Keywords:** supramolecular sensing, functional polymers, Zn-salphen building blocks, selective recognition

## Abstract

In this work, we present the synthesis of a novel Zn-Salphen complex containing an allyl group, which was used as building block in the further preparation of a new family of functional terpolymers. These polymers were obtained through radical co-polymerization with methyl metacrylate (MMA) and *n*-butyl acrylate (*n*BuA) in different ratios. The supramolecular recognition behavior of each polymer was evaluated via potentiometric measurements against selected anions in aqueous media. Interestingly, this proof of concept study shows that these systems were selective against only fluoride (F^−^) or both, fluoride and acetate (OAc^−^), by tailoring the relative content of Zn-Salphen monomer, thus making them a promising starting point for modular design of chemical sensors through straightforward synthetic approaches.

## 1. Introduction

Zn-Salphen complexes are compounds that can be synthesized by the condensation of *o*-phenylene diamine and salicylaldehyde derivatives, which, after coordination with Zn(II), form a wide range of imine-based complexes showing interesting and useful chemical features. Among these characteristics, their advantageous easy tuning properties through chemical functionalization of the aryl groups from the ligand part has facilitated their use in a wide range of applications: e.g., chemosensors for anions [1,2], organic photo-emitters in optoelectronics [3,4,5,6], and as catalysts [7,8,9]. 

With respect to their molecular geometry, Zn-Salphen complexes counterintuitively present a planar square N_2_O_2_ cavity after being coordinated to the (Lewis-acidic) Zn(II) center, where the geometry imposed by the Salphen ligand leaves a non-hindered vacant site for further supramolecular interactions with suitable Lewis-basic guests (e.g., via coordination, coulombic, etc.) [10]. In this regard, the possibility for further intermolecular coordination between the Zn center and O atoms, which belong to either the N_2_O_2_ moiety of additional Zn-Salphen complex molecules or N and/or O atoms that are present in other guest type molecules, has propelled the versatility of these kind of complexes. The latter has delivered access to their wide use as building blocks in self-assembled nanoarchitectures [11], auto-organized nanofibers [12], and many other different supramolecular host-guest systems [13,14,15]. This situation has inspired new strategies to develop a variety of supramolecular applications, such as self-assembled soft-matter [16,17,18,19], supramolecular sensors [20,21,22,23], and supramolecular catalysis [24,25,26].

As an example, we have previously reported on a new and simple strategy for synthesizing unsymmetrical Zn-Salphen complexes having, simultaneously, an aldimine moiety and a ketimine part [27], In this manner, the strategy allowed for structural functionalization by using salicylaldehydes with different functional groups. In that work, we used this approach to build a new library of modular Zn-Salphen compounds with tunable supramolecular capabilities. These tailored characteristics were further translated into the anisotropic self-assembly control of networked nanostructures in a polymer matrix that mimicked the neurons in a brain. Furthermore, we very recently exploited that same approach to develop hierarchically self-assembled polymer composite nanomaterials with promising optoelectronic applications [28].

However, the use of Zn-Salphen building blocks as a broadly applicable strategy for developing molecularly tunable materials in polymer science is still in its early stages [29]. In the literature, these building blocks have been incorporated into polymeric systems using two main strategies: supramolecular functionalization to obtain Zn-Salphen-composites [30,31,32] and homo-polymerization to produce covalently linked Zn-Salphen oligomers [33,34]. Nevertheless, to date, their potential as a supramolecular tool for tailoring the functional properties of more complex co-polymer materials has remained underexploited. This situation has resulted from the limited number of explored strategies to obtain unsymmetrical Zn-Salphen building blocks which in turn, could be further incorporated into larger, yet easily tunable multicomponent polymers, where the resulting materials could find direct applications in, for instance, customizable supramolecular sensing platforms.

**Figure 1:**
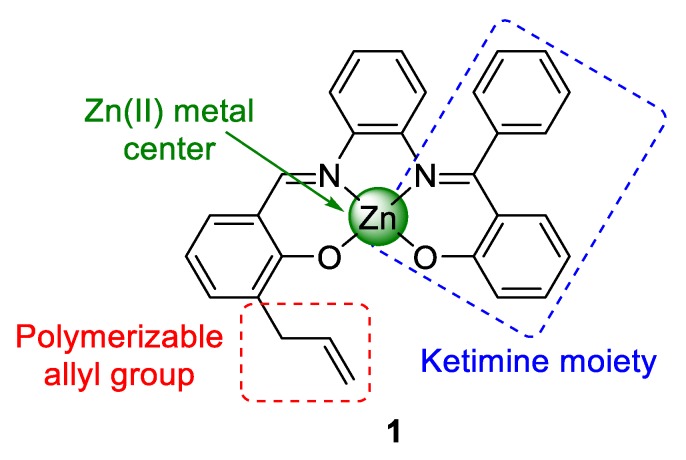
The Zn-Salphen complex **1**, functionalized with a polymerizable allyl group designed, synthesized, and characterized in this work.

Herein we report on the design, synthesis, and characterization of a novel unsymmetrical Lewis acidic Zn-Salphen complex, functionalized with an allyl moiety as a polymerizable group [35] and a di-aromatic ketimine moiety as a steric modulator (Figure 1). As a proof of concept, we demonstrate that the complex can be used in different ratios as an anion recognition building block during the synthesis of functional polymers via radical co-polymerization with a suitable fixed ratio of acrylic monomers *n*BuA and MMA. These acrylic monomers were selected as the major backbone components with a weight ratio of *n*BuA:MMA 8:2 according to the literature [36], where this combination showed optimal ion permeation capabilities in aqueous ion transfer studies using polymer films, while the material also demonstrated a proper combination of cohesiveness (derived from *n*BuA) and rigidity (delivered by MMA) during film preparation. Our new family of terpolymers was shown to be useful in the supramolecular sensing of anion guests, where the selectivity towards F^−^ and OAc^−^ anions could be easily tuned by the relative composition of functional building blocks. Interestingly, this terpolymer synthesis approach created the conditions to new venues in chemically tailored sensing platforms in a simplified manner.

## 2. Results and Discussion

**Scheme 1:**
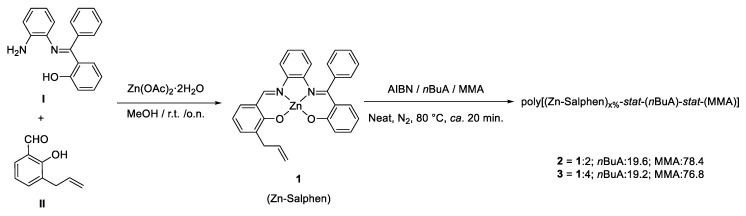
Synthetic route followed to obtain the building block Zn-Salphen 1 and its terpolymer counterparts, 2 and 3.

The unsymmetrical building block, Zn-Salphen complex **1** containing an allyl group, was synthesized and fully characterized to be further co-polymerized in different ratios by radical polymerization with MMA and *n*BuA, thus allowing access to a new Zn-Salfen-containing terpolymer family (Scheme 1). These new polymers (2 and 3) were successfully used in the supramolecular sensing of different guests (anions OAc^−^, F^−^, SCN^−^ and Glu^−^) with good selectivities.

### 2.1. Synthesis and Characterization of Zn-Salphen Complex, **1**

The synthesis of building block, Zn-Salphen complex 1 was performed following the procedure shown in Scheme 1. Ketimine I was reacted in situ with 1 equivalent of 2-hydroxy-3-allyl-benzaldehyde II in the presence of 1 equivalent of Zn(OAc)_2_·2H_2_O in MeOH and stirred overnight. The ^1^H NMR spectrum (Supplementary Information, Figure S1) indicates the obtaining of complex 1 where, at downfield (8.84 ppm), the characteristic signal of the CH of aldimine is present, and the 16 CH aromatic hydrogens were present in the region of 7.54–6.27 ppm, as expected. For the double bond, the CH signal is present at 6.07 ppm and CH_2_ signals at 5.12 and 5.00 ppm. For enantiotopic hydrogens of methylene, the signal is partially overlapped at 3.38 ppm with the signal of water present in DMSO-*d*_6_. In the ^13^C{^1^H} and 2D Heteronuclear Single Quantum Coherence (HSQC) NMR spectra (Figures S1 and S2), the characteristic signal of the quaternary carbon of ketimine, is present at 173.47 ppm. The characteristic signal of CH carbon of aldimine is present at 162.68 ppm. For the double bond, the CH signal is present at 138.00 ppm, and CH_2_ signal at 115.01 ppm. The carbon signal at 34.41 ppm corresponds to the CH_2_ of the methylene group adjacent to the double bond. HR-MS is in good agreement with the protonated molecular ion (See Experimental Section).

### 2.2. Synthesis and Characterization of Poly[(Zn-Salphen)_x%_-stat-(nBuA)-stat-(MMA)] Terpolymers 2 and 3

Zn-Salphen 1-containing terpolymers 2 and 3, namely poly[(Zn-Salphen)_2%_-*stat*-(*n*BuA)-*stat*-(MMA)] 2 and poly[(Zn-Salphen)_4%_-*stat*-(*n*BuA)-*stat*-(MMA)] 3 were synthesized by radical co-polymerization of *n*BuA and MMA varying the relative amount of Zn-Salphen 1 present therein (2% wt. for 2 and 4% wt. for 3), preserving the nominal weight ratios of *n*BuA:MMA = 8:2. The synthesis was performed under inert atmosphere without solvent, at 80 °C for 20 min in presence of AIBN as initiator, affording the terpolymers 2 and 3 in good yields (98% and 97% respectively). In the ^1^H NMR spectra recorded in CDCl_3_ (Figures S3 and S4), broad signals—a typical behavior in spectra of polymers—for the different aliphatic hydrogens of polymers 2 and 3 are present in the regions 1.75–2.5 ppm and 3.5–4.25 ppm. It is important to remark that these signals are also respectively present in the homologue blank copolymer 4 (*n*BuA:MMA = 8:2) (see Figure S5). It is interesting to mention the presence of some very small sets of olefinic signals around 5 ppm, some aromatic signals between 6.6–7.7 ppm, and the characteristic singlet at 8.33 ppm corresponding to the CH of the aldimine, belonging to 1 in the spectra obtained for 2 and 3. The simultaneous occurrence of the later signals, whose intensities increase with an increase of the nominal ratio of 1, indicating the presence of 1 in the terpolymer counterparts 2 and 3. On the contrary, the same are absent in the blank copolymer 4, as expected. The signals corresponding to olefinic hydrogens appearing around 5.5–6.5 ppm (Figures S3 and S4) correspond to some occluded *n*BuA monomers, which could be further removed when solid-state films are prepared for supramolecular recognition studies (see Experimental Section).

For comparison, it is important to mention here that the weight-average molecular weight (${\bar{M}}_{w}$) and number-average molecular weight (${\bar{M}}_{n}$) of the blank copolymer 4 determined by Gel permeation chromatography (GPC) was ca. 84,400 g/mol and ca. 21,200 g/mol, respectively, with a number-average degree of polymerization (${\bar{X}}_{n}$) of 175. However, the incorporation of the Zn-Salphen 1 building block in terpolymers 2 and 3 displaced their ${\bar{M}}_{w}$ to lower values, i.e., to ca. 71,000 g/mol and ca. 81,200 g/mol, respectively, while ${\bar{M}}_{n}$ together with ${\bar{X}}_{n}$ roughly followed the opposite trend: cf. ${\bar{M}}_{n}$ = 21,000 g/mol and ${\bar{X}}_{n}$ = 169 vs. ${\bar{M}}_{n}$ = 48,000 g/mol and ${\bar{X}}_{n}$ = 384, respectively, for 2 and 3. Interestingly, the increase in content of building block 1 in terpolymers 2 and 3 (if compared with copolymer 4) facilitated control of the molecular-weight dispersity (*Ð*_M_) from a non-uniform molar mass distribution at 0% wt. (content of Zn-Salphen) to a more uniform statistical distribution thereof upon increasing such monomer to 2% wt. and 4% wt. In other words, the higher the content of Zn-Salphen building block 1 in the polymer composition, the lower the dispersity value. This can be attributed to a synergistic control on the radical polymerization mechanism carried out by the Zn-salphen component. Such an effect might be mechanistically driven by either one of the following factors or by a combination thereof: (i) initiator radical entrapment by the Zn center [37] and/or (ii) degradative chain transferring [38] through the allyl moiety during chain growth. Regardless of the mechanistic manifold underlying such trend, a simultaneous decrease in dispersity concerted with an increase in degree of polymerization, should be a result of a constrained number of available reactive radicals at the initiation step (the lower the number of starting radicals, the larger the polymer chains), together with a limited occurrence of multiple termination reaction alternatives (e.g., if termination is, for instance, mostly displaced to second order radical-radical reactions such as combination or disproportionation, a lower dispersity should arise). The last scenario is compatible with either mechanisms i or ii. However, which of these possibilities predominates remains as an open question, and, as they fall out of the scope of this work, future kinetic studies will be required to unravel the molecular pathways that rule this interesting phenomenon. In Table 1, GPC information is summarized.

Thermogravimetric analyses (TGA) determined that all the polymers 2–4 were highly stable up to ca. 270 °C, with accumulated mass losses of as low as 3% wt. at this temperature for the worst case scenario (terpolymer 3), while all of them remained relatively stable up to ca. 300 °C, with onset decomposition temperatures (T_o_) starting between 318 °C and 345 °C and final decomposition temperatures (T_f_) appearing up to ca. 390 °C (See Experimental Section and Figure S6). It is interesting to remark that, in comparison with blank copolymer 4, the Zn-Salphen building block exerted a narrowing effect on the range comprised between T_o_ and T_f_, where the inclusion of the monomer dropped such range in ca. 30 °C. A closer inspection of the curve patterns in the later range of temperatures for polymers 2 and 3 shows an absence of individually stepped weight losses together with a minor impact on the first derivative peak temperature (T_p_) if compared with the blank 4—I.e., T_p_ is displaced from 378 °C to 373 °C, when the content of 1 is increased from 0% wt. to 4% wt. The previous effect occurring together with the narrower T_o_-T_f_ interval for the polymer with the higher content in 1, reveals that major decomposition occurs in one event, which in turn suggests that component 1 should be part of the same macromolecular structure, rather than a separate component phase in the acrylic matrix. In addition, both the residual mass after decomposition and the mass lost throughout the decomposition temperature range followed the behavior expected for increasing the Zn-Salphen 1 content, where the residue increased from 9% wt. for 4 up to 17% wt. for 3, and the lost weight decreased from 95% wt. for 4 to 85% wt. for 3. In other words, the higher the amount of the functional monomer, the higher the residual mass and the lower the mass loss. The whole scenario is in good agreement with previously reported metal-Schiff base complex-containing polymers, as these components allow one to generate high temperature-resistant materials [33]. On the other hand, Differential scanning calorimetry (DSC) showed that the 2% wt. in Zn-Salphen content for terpolymer 2 did not cause a significant influence on the glass transition temperature measured during the heating cycle, since both the terpolymer and its non-containing counterpart 4 yielded a T_g_ of ca. −28 °C (See Experimental Section and Figure S7). However, by rising the amount of the functional monomer 1 to 4% wt. in terpolymer 3, T_g_ was displaced to ca. −51 °C. This phenomenon could be explained by a plasticizing effect carried out by the Zn-Salphen component, which should be triggered when the number of 1 monomer units in a terpolymer chain are present above a threshold value beyond 2% wt. In supramolecular terms, the formed could be explained by a hampering effect from the bulky Zn-Salphen moiety on the inter-chain (i.e., polymer-polymer) van der Waals interactions which are, in turn, the forces ruling cohesiveness in these materials. Either in the absence or the presence of Zn-Salphen monomer, these cohesive forces are mostly driven by the *n*-butyl moieties in the *n*BuA monomer (major component), together with the predominantly aliphatic backbone. These interactions should be mainly responsible for keeping polymer chains at the vitreous state (glassy amorphous state), where, if the functional monomer composition threshold is surpassed, as in polymer 3 with a 4% wt. of 1, the aforementioned steric effects may eventually become significant, thereby decreasing the energy barrier needed to have a transition to the viscous state upon heating. To this point, it is important to highlight that the later effect played a major role in selectivity modulation during our supramolecular recognition studies, as will be discussed in the Section 2.3. In our set of polymers, all of them statistically have the same major backbone (*n*BuA and MMA domains), with minor compositional variations in the Zn-Salphen component. For a system such as the one herein studied, the possibility to decrease the glass transition temperature by slightly rising one of the components allows one to increase the solid-state polymer free volume at temperatures above T_g_, if compared to those polymer counterparts with higher T_g_ values. In other words, and in wide accordance with the literature [39], increasing the polymer free volume at ambient conditions is possible as long as its T_g_ is dropped, which is the case when Zn-Salphen 1 content is increased from 2% wt. in polymer 2 to 4% wt. in polymer 3. For a solid-state polymer system bearing supramolecular host units in its chain (ion recognition sites), a rise in free volume would open the opportunity for both smaller and larger diffusible guests (target ions) to more easily permeate across the solid phase. As a consequence, further interaction of target ions with more accessible ion recognition sites via host-guest chemistry is not only facilitated but also creates additional competitive scenarios where the short- and mid-range attractive and repulsive supramolecular interactions between the rest of the backbone and the guest could potentially provide differences in selectivity.

### 2.3. Supramolecular Recognition Studies

As a proof of concept, the supramolecular recognition behavior of the polymers against selected Lewis-basic anions was assessed by determining the potentiometric response that testing films prepared from each system were able to produce in aqueous media. In the evaluation, a more pronounced absolute change in the monitored electromotive force (EMF) values vs. the anions represented a higher permeation through the polymer films deposited on the evaluation platform. If such permeation is mainly driven by selective supramolecular interactions (e.g., coordinative and ion-dipole interactions towards a recognition site incorporated therein), then a discriminated response could arise when testing the specimens against anions of a different nature. In this sense, potentiometry was chosen as a straightforward monitoring tool for assessing passive anion permeation from the aqueous phase to the solid-state polymer phase (the testing films) because of its easy implementation if an ion-to-electron electro-conducting transducer (such as indium-tin oxide, namely ITO, our evaluation platform) is adequately coated with the material tested. On the one hand, if the aqueous concentration of an anion is increased, and this anion is able to spontaneously permeate into a tested film that is exposed to these conditions, then an increase in the number of such charged species inside the film would occur via the establishment of an aqueous/polymer biphasic partition equilibrium. In these conditions, the underlying transducer would translate such variation into a change in EMF response that is recorded by a high-impedance potentiometer. On the other hand, if the anion under study does not favorably permeate into the polymer solid phase (i.e., it has a lack of a partition equilibrium), then an absence in EMF response would be observed. In either case, displacing such partition equilibrium towards the polymer phase is possible as long as the anion finds a suitable balance of supramolecular attractive forces inside the matrix, together with a minor impact from the repulsive interactions present therein. This can be carried out either by the incorporation of host-guest recognition sites in the solid-state phase or by incorporation of compatible functional groups in the polymer backbone.

**Figure 2:**
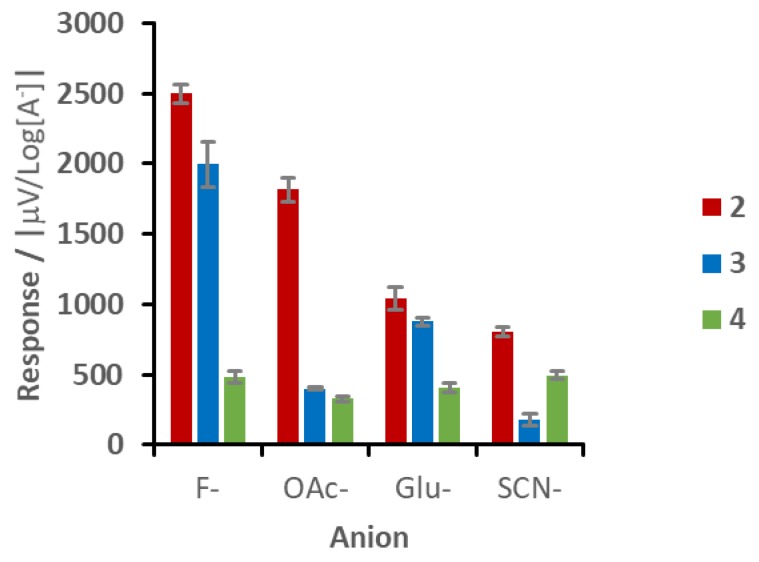
Polymers synthesized (**2**–**4**) and their response (absolute numbers) against selected anions of fluoride, acetate, gluconate and thiocyanate, as evaluated by potentiometry.

The results showed a direct influence of the functional monomer 1 content (cf. 2% wt. vs. 4% wt.) on the Lewis-basic anions that could favorably interact with the Lewis-acidic Zn centers in the polymers (see Figure 2). On the one hand, terpolymer 2 afforded a noticeable response against the four anions if compared with blank copolymer counterpart 4. In such a case, the degree of each response varied depending upon which anion was eventually tested, where the most significant signals were attained for both fluoride and acetate and then followed by gluconate and thiocyanate, in that order (i.e., F^−^ ≥ OAc^−^ > Glu^−^ > SCN^−^). It is critical to remark that the difference in response between fluoride and acetate did not exceed ca. 30%, but both anions caused responses which were markedly higher (between ca. 200%–300%) than the values for the other two anions. On the other hand, terpolymer 3 showed a similar response degree against fluoride if compared with the polymer 2 counterpart (a difference of ca. 20%). Nonetheless, such a polymer yielded totally different scenario for the rest of the anions. In polymer 3, the decreasing order in the signals followed the trend F^−^ > Glu^−^ > OAc^−^ > SCN^−^, since the response for gluconate was significantly lower than the one for fluoride (more than twofold) but still higher than the response in polymer 4 and of the same order as polymer 2. However, in polymer 3, the respective values for acetate and thiocyanate were either in the same order or lower than the corresponding measurements obtained with blank copolymer 4.

**Figure 3:**
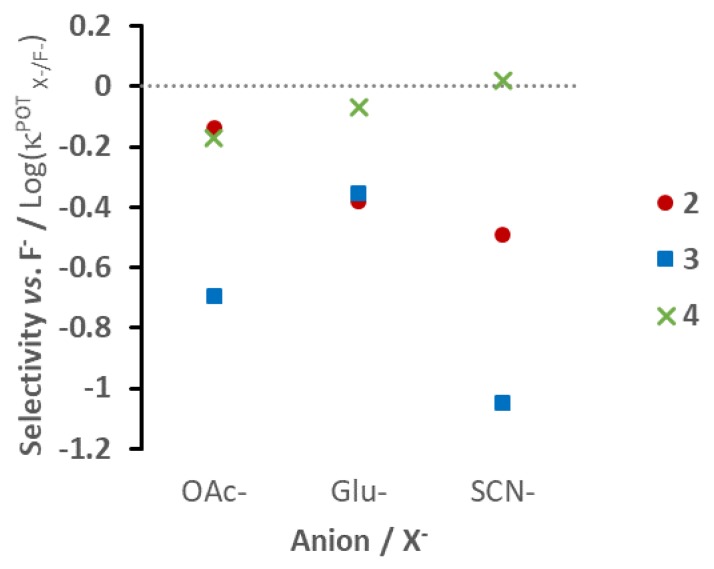
Relative selectivity, Log(κ^POT^_X-/F-_), obtained with each polymer (**2**–**4**), estimated for F^−^ vs. each anion X^−^, where the gray horizontal dashed line is the reference value for the fluoride target guest.

Given that the highest responses in terpolymers 2 and 3 occurred when these systems were exposed to a fluoride anion, we determined—as a further step—the selectivity values of the polymers **2**–**4** using this ion as the target guest and the remaining three ions as interfering anions (see Figure 3). The results showed that for terpolymer 2, selectivity between fluoride and acetate was logarithmically negligible. However, gluconate and thiocyanate afforded the highest discrimination values, since the anion SCN^−^ provided the most marked selectivity with fluoride (a higher distance from the F^−^ reference). Contrarily, terpolymer 3 showed that a higher selectivity was achievable for fluoride vs. the whole set of interfering anions when the Zn-Salphen monomer content was raised from 2% wt. to 4% wt. In this case, the highest selectivity was also achieved against SCN^−^. Nonetheless, gluconate showed the closest value to the reference guest, instead of acetate (as in 2). As a reference, a similar analysis carried out for blank copolymer 4 showed that no selectivity was achieved for F^−^ against thiocyanate, whereas a minor selectivity arose vs. gluconate and a slightly more pronounced discrimination occured vs. acetate. However, this value is still logarithmically negligible.

In this regard, the differential response triggered by these anions as a result of a change in monomer 1 composition in the range comprised of 0% wt. to 4% wt. illustrates an important fact, which is directly related to a synergistic participation of the whole set of supramolecular interactions that are viable in each polymer/anion scenario. To simplify, short-range and mid-range supramolecular interactions, which can be either attractive or repulsive, are responsible for this modulated response. For example, the increased selectivity of polymer 2 for F^−^ and OAc^−^ (Figure 3, red circles) or polymer 3 for only F^−^ over the rest of the anions (Figure 3, blue squares) might both be a result of its predominance in short-range attractive interactions, such as coordination bond formation between the Zn center (a “hard” Lewis acid), and either the fluoride ion or the oxygen atoms in acetate (two “hard” Lewis bases). Although this scenario is also hypothetically plausible for gluconate (a guest with one carboxylate moiety in its structure) in polymer 2, its larger guest-size would potentially represent a limitation for its unhindered migration throughout the polymer matrix to the Zn-Salphen moieties at 2% wt., which could then explain the higher discrimination observed for 2 against this anion, occurring together with a higher response of the system when the smaller acetate anion is otherwise analyzed. This limiting factor is, in part, mitigated when Zn-Salphen content is increased to 4% wt. in 3. As demonstrated by DSC and discussed in the previous Section 2.2, the major role played by the bulkiness of monomer 1—to simultaneously decrease the inter-chain van der Waals attractive forces and increase the solid-state polymer free volume in 3—creates an opportunity for the larger gluconate anion to find permeation paths through the film, aided by favorable mid-range attractive dipole–dipole interactions between the gluconate –OH groups and the acrylic carbonyl moieties in the polymer backbone. In contrast, the absence of these dipolar hydroxyl moieties in acetate, together with a facilitated role of the mid-range repulsive anion–dipole interactions between the carboxylate moiety in acetate and the partially negative acrylic carbonyl portions in the polymer chain, represent a competitive disadvantage for this anion once inside terpolymer 3. In this case, the permeation of acetate through the polymer 3 phase to find its further coordination with Zn-centers is jeopardized, in spite of its reduced molecular size if compared with gluconate; this situation is illustrated in Figure 3 by the lower selectivity for OAc^−^ as compared with Glu^−^ for polymer 3 and the opposite trend for polymer 2. In other words, under a higher polymer free volume scenario, such as the one imposed by Zn-Salphen content 4% wt. (polymer 3), both carboxylated guests, acetate and gluconate, could hypothetically find facilitated access across the tested films towards the recognition sites, regardless of their difference in size. Once these anions are below a critical distance from the recognition sites, then the short-range attractive carboxylate-Zn coordination interactions would theoretically act as the major driving force for displacing partition equilibria towards the polymer phase. However, in reality, the presence of five –OH substituents in the gluconate structure gives access to additional non-selective attractive supramolecular interactions between this guest and the polymer backbone in 3. Conversely, the only presence of the carboxylate moiety in the acetate is not enough to overcome the repulsive barrier imposed by the high number of partially negative ester groups throughout the polymer chains under a higher free volume scenario in the same polymer 3. This effect was also observed, although to a lesser extent, in the absence of the Zn-Salphen monomer for polymer 4; in Figure 3 (green crosses), the higher discrimination observed for OAc^−^ represents a decreased permeation to the polymer phase, while the lower selectivity yielded by Glu^−^ means that an increased migration of this anion into the polymer film occurs. On the other hand, for the ambidentate anion SCN^−^, which could in theory also participate in the coordination to the Zn center by the “harder” N atom, the results demonstrated that this anion does not trigger any potentiometric response in either 2 or 3, which consequently means that a very limited amount of thiocyanate is permeated to the polymer phase. The latter could be similarly explained by mid-range repulsive ion-dipole interactions between the anion and the partially negative acrylic carbonyl portions in the terpolymers 2 and 3. This scenario would also explain, in general terms, the very limited potentiometric response triggered by all the anions in the absence of monomer 1, such as in blank copolymer 4.

## 3. Experimental Section

### 3.1. Materials and Methods

All starting compounds and solvents were purchased from Sigma-Aldrich Co. (Saint-Louis, MI, USA) and used without further purification, unless otherwise stated. Compound (*E*)-2-(((2-aminophenyl)imino)(phenyl)methyl) phenol (ketimine, I) was prepared using a previously reported methodology [40]. All nuclear magnetic resonance (NMR) measurements were carried out on a Varian VNMRS 400 MHz spectrometer (Varian Inc., Palo Alto, CA, USA) at ambient temperature unless otherwise stated, and chemical shifts are given in parts per million versus TMS. Thermogravimetric analyses (TGA) were performed under nitrogen flow with a ramp of 10 °C/min on a Perkin Elmer TGA4000 apparatus (Perkin Elmer Inc., Waltham, MA, USA); starting temperature 30 °C, final temperature 460 °C. Differential scanning calorimetry (DSC) measurements were performed under nitrogen atmosphere with a ramp of 10 °C/min (unless otherwise stated) on a Mettler Toledo DSC1 calorimeter (Mettler-Toledo, LLC, Columbus, OH, USA) at Unidad de Servicios de Apoyo a la Investigación y a la Industria (USAII) of the School of Chemistry–UNAM; conditioning cycles: r.t. to 100 °C, isothermal at 100 °C for 10 min, 100 °C to −100 °C, isothermal at −100 °C for 10 min; T_g_ measurement cycle: −100 °C to 250 °C. High resolution mass spectrometric data (HRMS) were obtained with an Agilent 6530 QTOF spectrometer (Agilent Technologies, Santa Clara, CA, USA). Gel permeation chromatography (GPC) determinations were made on an Agilent Technologies 1290 infinity UHPLC with a Q-TOF detector 6530 DUAL AJ ESI (Santa Clara, CA, USA) using chromatography-grade tetrahydrofuran as solvent at Unidad de Servicios para la Industria Petrolera (USIP) of the School of Chemistry–UNAM.

Polyethylene terephthalate (PET) films coated with indium-tin oxide (ITO) with surface resistivity 60 Ω/sq and a coating thickness of 1300 Å were purchased from Sigma-Aldrich. Deionized water for the potentiometric measurements was obtained from a Thermo Scientific Barnstead Nanopure deionization apparatus (Thermo Fisher Scientific Inc., Waltham, MA, USA). Potentiometric measurements were taken with a model EMF-16 high-impedance potentiometer (Lawson Labs, Inc., Malvern, PA, USA) with 8 independent channels. Electromotive force data was automatically recorded by means of the Data Acquisition and Instrument Controller (L-EMF DAQ 3.0) software also provided by Lawson Labs, Inc. All the potentiometric determinations were taken using an Ag/AgCl/KCl (3 M) double junction reference electrode containing a 1 M KCl electrolyte bridge (Mettler Toledo InLab Reference, Mettler-Toledo, LLC, Columbus, OH, USA).

### 3.2. Synthesis of Zn-Salphen Complex, **1**

To a solution of ketimine I (585 mg, 2.03 mmol) in 10 mL of MeOH we added a solution of 3-allyl-2-hydroxybenzaldehyde II (0.35 mL, 2.30 mmol) and Zn(OAc)_2_·2H_2_O (468 mg, 2.13 mmol) in 5 mL of MeOH. The reaction was stirred for 18 h and the yellow solid was filtered and washed with MeOH (708 mg, 70%). ^1^H NMR (400 MHz, DMSO-*d*_6_): *δ* = 8.84 (s, 1H), 7.54 (d, *J* = 8 Hz, 1H), 7.39–7.40 (m, 3H), 7.27 (dd, *J* = 7.9, 1.9 Hz, 1H), 7.24–7.21 (m, 2H), 7.18–7.12 (m, 2H), 7.09 (t, *J* = 7.7 Hz, 1H), 6.87 (dd, *J* = 8.3, 1.9 Hz, 1H) 6.81 (t, *J* = 8 Hz, 1H), 6.77 (d, *J* = 8.4 Hz, 1H), 6.50–6.44 (m, 2H), 6.27 (t, *J* = 7.2 Hz, 1H), 6.07 (ddt, *J* = 13.8, 9.9, 6.9 Hz, 1H), 5.12 (d, *J* = 17 Hz, 1H), 5.00 (d, *J* = 9.9 Hz, 1H), 3.38 (d, *J* = 6.8 Hz, 2H) ppm. ^13^C{^1^H} NMR (100 MHz, DMSO-*d*_6_), δ = 173.44, 170.15, 162.68, 139.97, 139.58, 138.00, 136.84, 134.29, 134.19, 133.19, 132.36, 128.71, 128.42, 128.20, 125.70, 125.37, 124.23, 123.16, 120.46, 118.59, 116.83, 115.01, 112.47, 112.14, 34.41. ppm. HRMS (ESI-QTOF) m/z found 495.10576 ([M+H]), C_29_H_23_N_2_O_2_Zn requires 495.10510. For ^1^H NMR, ^13^C{^1^H}, and HSQC spectra see Supplementary Figures S1 and S2.

### 3.3. Solvent Free Radical Polymerization

Under an N_2_ atmosphere, a mixture of the monomer Zn-Salphen complex **1**, *n*BuA, and MMA in different ratios was homogenized at room temperature and N_2_ was additionally bubbled through the solution for 5 min. Then, 10 mg of azobisisobutyronitrile (AIBN) were added. The reaction mixture was stirred for 20 min at 80 °C, and the resulting polymer was dissolved in CHCl_3_ and solvent was evaporated under a high vacuum to eliminate volatile *n*BuA and MMA monomers.

For poly[(Zn-Salphen)_2%_-*stat*-(*n*BuA)-*stat*-(MMA)], 2. Mass ratio (Zn-Salphen):*n*BuA:MMA = 2:78.4:19.6 (8:2 nominal for *n*BuA:MMA respectively). Transparent yellow highly viscous liquid (98%). ${\bar{M}}_{n}$, ${\bar{M}}_{w}$, ${\bar{M}}_{v}$, ${\bar{M}}_{z}$ (g mol^−1^, GPC): 20887, 71029, 175060, 207234. T_d_ (TGA): T_o_ = 345 °C, T_f_ = 390 °C, T_p_ = 378.3 °C, loss 92%, residue 10%. T_g_ (DSC): –28.5 °C. For ^1^H NMR spectrum see Supplementary Figure S3.

For poly[(Zn-Salphen)_4%_-*stat*-(*n*BuA)-*stat*-(MMA)], 3. Mass ratio (Zn-Salphen):*n*BuA:MMA = 4:76.8:19.2 (8:2 nominal for *n*BuA:MMA respectively). Transparent yellow highly viscous liquid (97%). ${\bar{M}}_{n}$, ${\bar{M}}_{w}$, ${\bar{M}}_{v}$, ${\bar{M}}_{z}$ (g mol^−1^, GPC): 48103, 81197, 126498, 139350. T_d_ (TGA): T_o_ = 338 °C, T_f_ = 390 °C, T_p_ = 373 °C, loss 85%, residue at T_f_ 17%. T_g_ (DSC): -51.6 °C. For ^1^H NMR spectrum see Supplementary Figure S4.

For blank copolymer poly[(*n*BuA)-*stat*-(MMA)], 4. Mass ratio *n*BuA:MMA = 8:2. Transparent colorless highly viscous liquid (99%). ${\bar{M}}_{n}$, ${\bar{M}}_{w}$, ${\bar{M}}_{v}$, ${\bar{M}}_{z}$ (g mol^−1^, GPC): 21194, 84378, 224387, 266924. T_d_ (TGA): T_o_ = 318 °C, T_f_ = 393 °C, T_p_ = 378 °C, loss 95%, residue at T_f_ 9%. T_g_ (DSC): -28.6 °C. For ^1^H NMR spectrum see Supplementary Figure S5.

### 3.4. Supramolecular Recognition Studies

#### 3.4.1. Preparation of the Evaluation Platforms

In a typical experiment, testing film deposition over ITO (0.05 cm^2^) was carried out following the doctor blade’s technique, using a solution of 20% *m*/*v* of the corresponding polymer **2**–**4** containing (6 × 10^−3^)% *m*/*v* of tetrabutylammonium hexafluorophosphate as an inert lipophilic counterion in CHCl_3_. Then, the deposited films were dried for 24 h under vacuum, thoroughly washed with de-ionized water, and dried again prior to further evaluation to guarantee that the solvent and remaining traces of monomers *n*BuA/MMA were completely removed. The thickness of the testing films was controlled through this process to a final value of 50 μm in the solid state using variable deposition speed, blade height, and sample volume depositing as needed. These films were mechanically stable for at least four weeks before testing, whereas the deposition technique allowed for easily prepared homogeneous surfaces.

#### 3.4.2. Analytical Procedure

Supramolecular recognition assessments were carried out using the zero-current potentiometry technique, where the selectivity and response of the polymers vs. different anions was followed by measuring the electromotive force (EMF) against anion concentration. All the EMF measurements were taken in a cell containing 50 mL of a KNO_3_ background solution with ionic strength fixed to 10 mM, at a neutral pH, 25 °C, stirred at 300 rpm, and a previous stabilization period of 30 min. The changes in EMF were automatically measured by adding concentrations of the anion under study (F^−^, OAc^−^, SCN^−^, Glu^−^) in a stepwise mode (10^−12^ mol/L to 10^−4^ mol/L in the cell) and following the variations on the recorded values. The dilution was corrected for all the stepwise concentration experiments. K^+^ was used as a counterion for all the anion stock solutions used. The stock solutions (1 mol/L) used in stepwise additions were prepared by diluting the required mass of the salt’s potassium fluoride, potassium acetate, potassium thiocyanate, and monopotassium gluconate, in 100 mL of the aforementioned KNO_3_ background solution, and the pH was adjusted to neutral.

## 4. Conclusions

In conclusion, the present work shows that a straightforward modulation of the supramolecular recognition behavior of a functional polymer could be achieved by the molecular design of a polymerizable Zn-Salphen monomer and its co-polymerization at different ratios with an acrylic backbone. The origin of such tailored recognition behavior is mainly an interplay between attractive host-guest interactions and either repulsive or attractive guest-polymer backbone interactions, depending on the nature of the guest, together with an increased polymer free volume when T_g_ is decreased by the bulky Zn-Salphen component. This situation paves the way for more simplified strategies in chemical sensor design, departing from one single functional monomer that only depends upon the composition, thereof.

## Figures and Tables

**Table 1:** Average molecular weights (${\bar{M}}_{w}$, ${\bar{M}}_{n}$), molecular-weight dispersities (*Ð*_M_) and number-average degrees of polymerization (${\bar{X}}_{n}$) for polymers 2–4 determined by Gel permeation chromatography (GPC).

Copolymer	${\bar{M}}_{w}$	${\bar{M}}_{n}$	*Ð*_M_	${\bar{X}}_{n}$
**2**	71,029	20,887	3.4	169
**3**	81,197	48,103	1.7	384
**4**	84,378	21,194	4.0	175

## Supplementary Materials

The following are available online at https://www.mdpi.com/1420-3049/24/12/2245/s1, Figure S1: ^1^H and ^13^C{^1^H} NMR spectra of 1. Figure S2: HSQC NMR spectrum of 1. Figure S3: ^1^H NMR spectrum of 2. Figure S4: ^1^H NMR spectrum of 3. Figure S5: ^1^H NMR spectrum of 4. Figure S6: TGA of polymers **2**–**4**. Figure S7: DSC of polymers **2**–**4**.
