# Supplementary material for: Synthesis of a Novel Zn-Salphen Building Block and Its Acrylic Terpolymer Counterparts as Tunable Supramolecular Recognition Systems
Source: Molecules. 2019 Jun 15;24(12):2245. doi: 10.3390/molecules24122245 (PMC6631749; doi:10.3390/molecules24122245)
Supplement: Supplementary file 1 [file molecules-24-02245-s001.pdf]

Supplementary Information for:

Synthesis of a novel Zn-Salphen building block and its acrylic terpolymer counterparts as tunable supramolecular recognition systems

Gustavo A. Zelada-Guillén [1,*], Ana B. Cuéllar-Sánchez [1], Margarita Romero-Ávila [1] and Martha V. Escárcega-Bobadilla [1,*]

[1] School of Chemistry, National Autonomous University of Mexico (UNAM), Circuito Escolar s/n, Ciudad Universitaria, 04510 Mexico City, Mexico

***Correspondence**: g.zelada@unam.mx (G.A.Z.-G.); mesbo@unam.mx (M.V.E.-B.)

Table of contents:

Page S2: **Figure S1**. ^{1}H and $^{13}C\{^{1}H\}$ NMR spectra of **1**
Page S3: **Figure S2**. HSQC NMR spectrum of **1**
Page S4: **Figure S3**. ^{1}H NMR spectrum of **2**
Page S5: **Figure S4**. ^{1}H NMR spectrum of **3**
Page S6: **Figure S5**. ^{1}H NMR spectrum of **4**
Page S7: **Figure S6**. TGA curves of **2** – **4**
Page S8: **Figure S7**. DSC curves of **2** – **4**

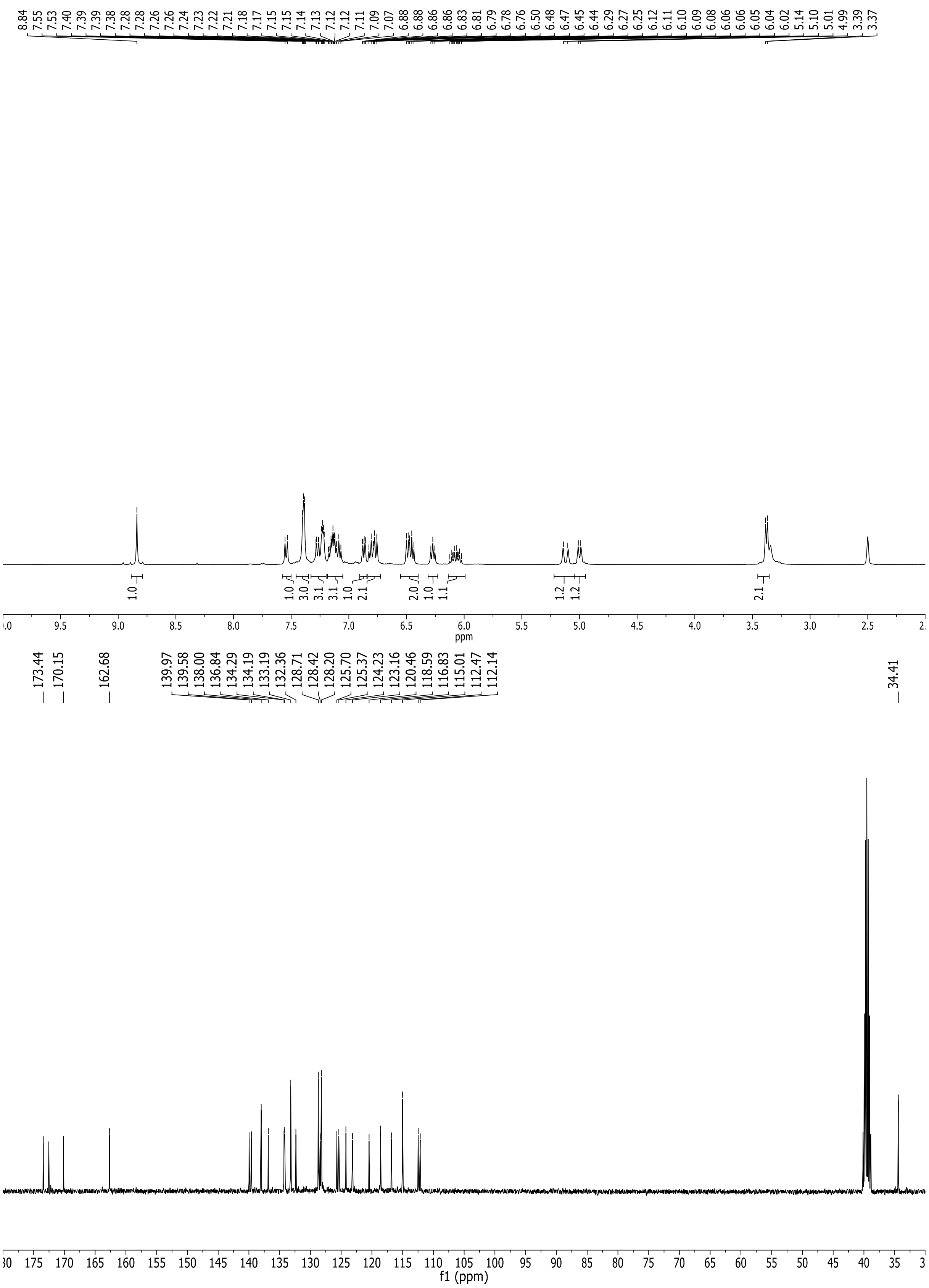

Figure S1. Top: ^{1}H NMR spectrum, and bottom: $^{13}C\{^{1}H\}$ of **1** in DMSO-d_6 at 298 K.

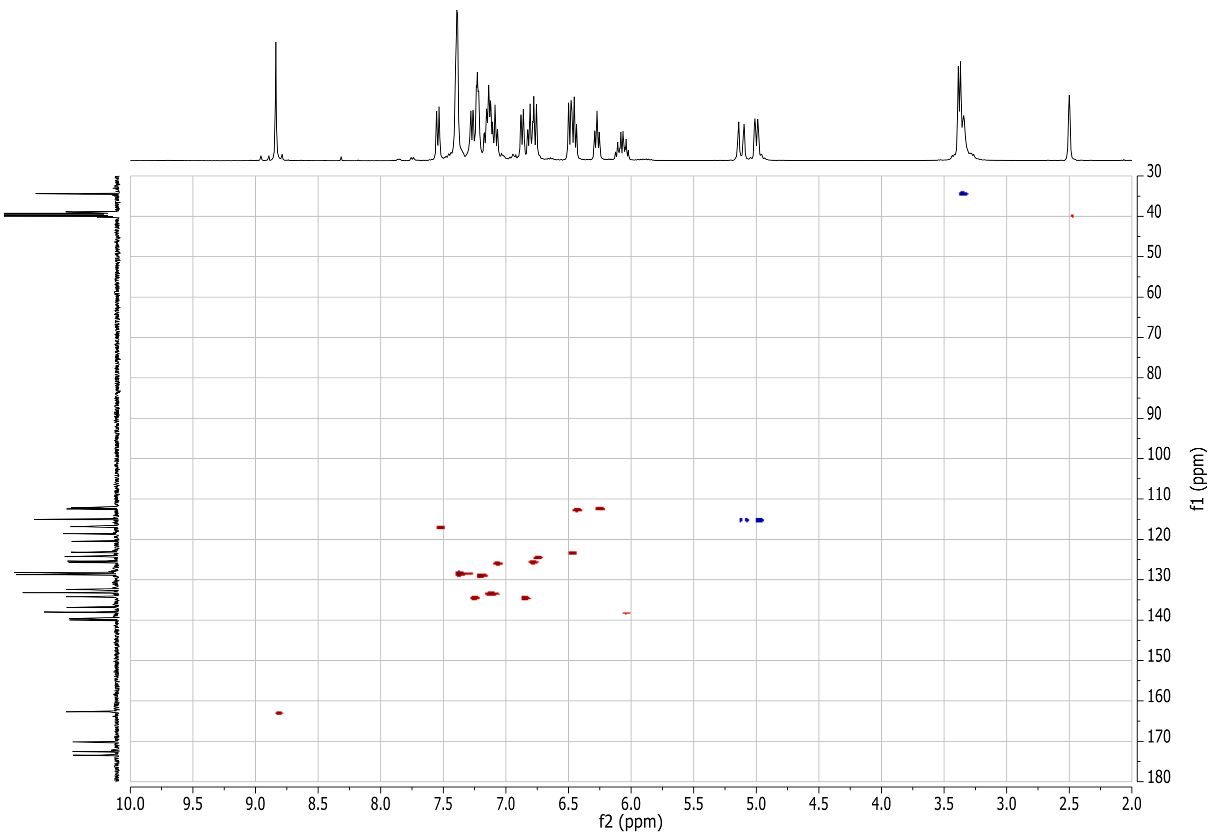

Figure S2. HSQC NMR spectrum of **1** in DMSO-d_6 at 298 K.

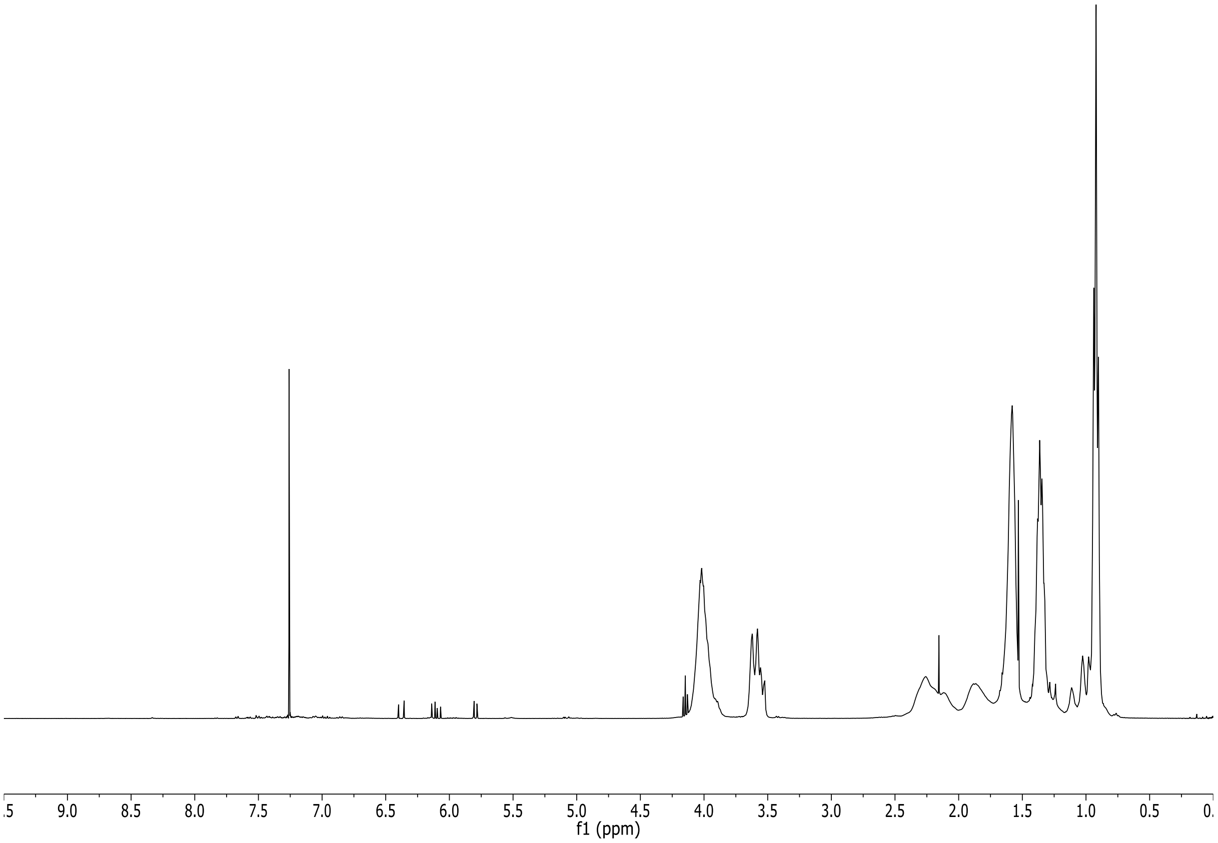

Figure S3. ^{1}H NMR spectrum of **2** in $CDCl_3$ at 298 K.

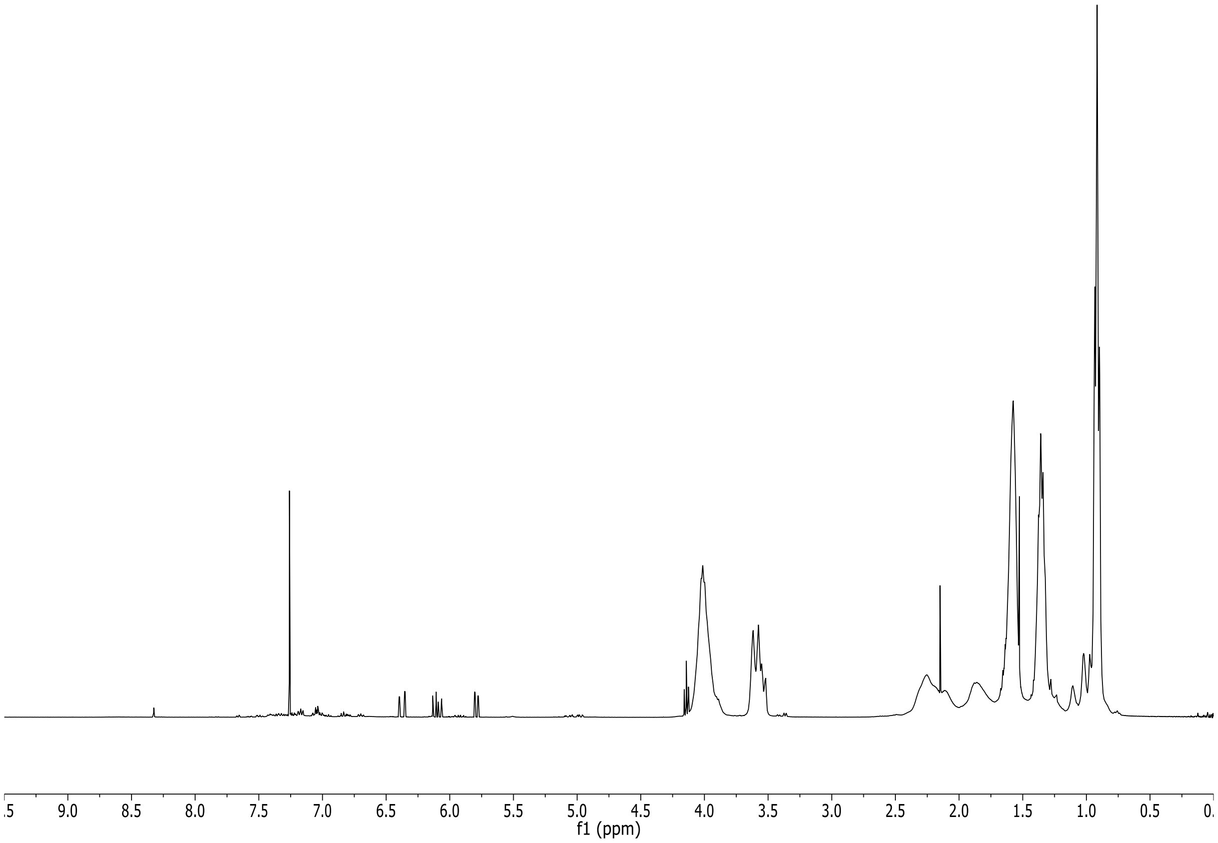

Figure S4. ^{1}H NMR spectrum of **3** in $CDCl_3$ at 298 K.

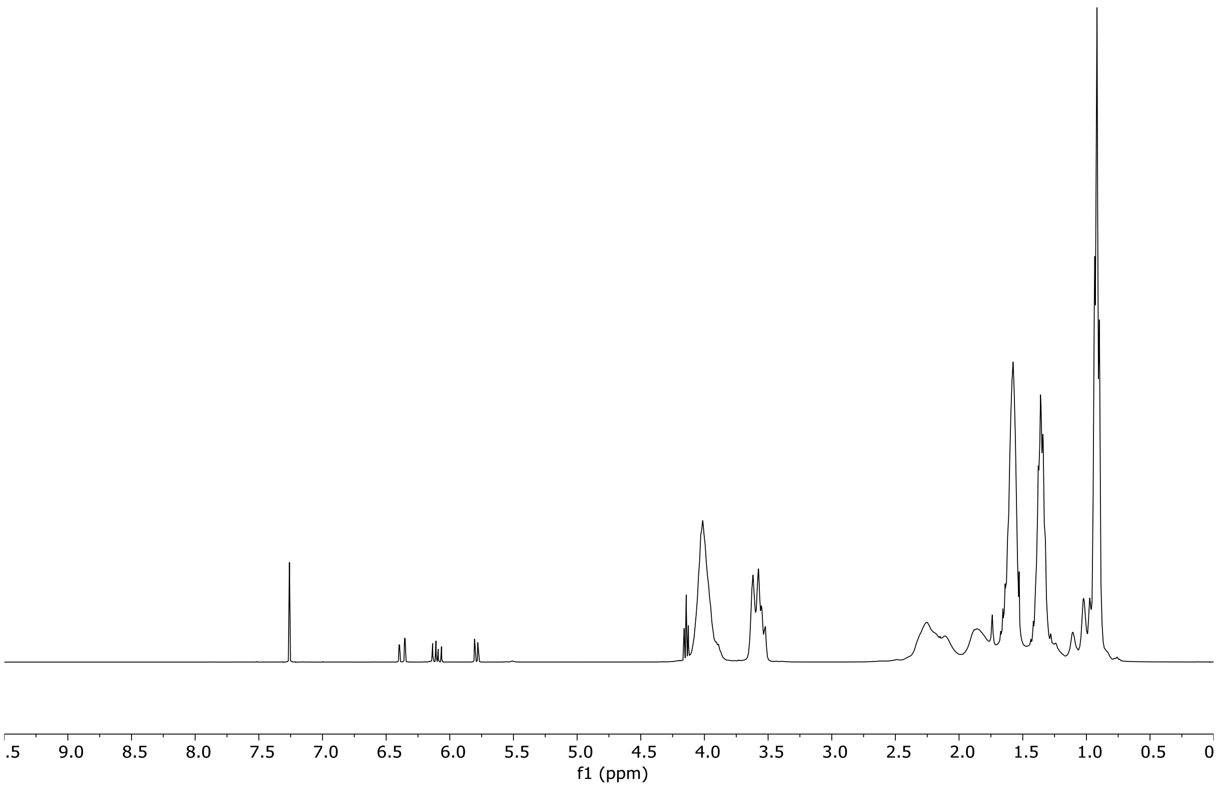

Figure S5. ^{1}H NMR spectrum of **4** in $CDCl_3$ at 298 K.

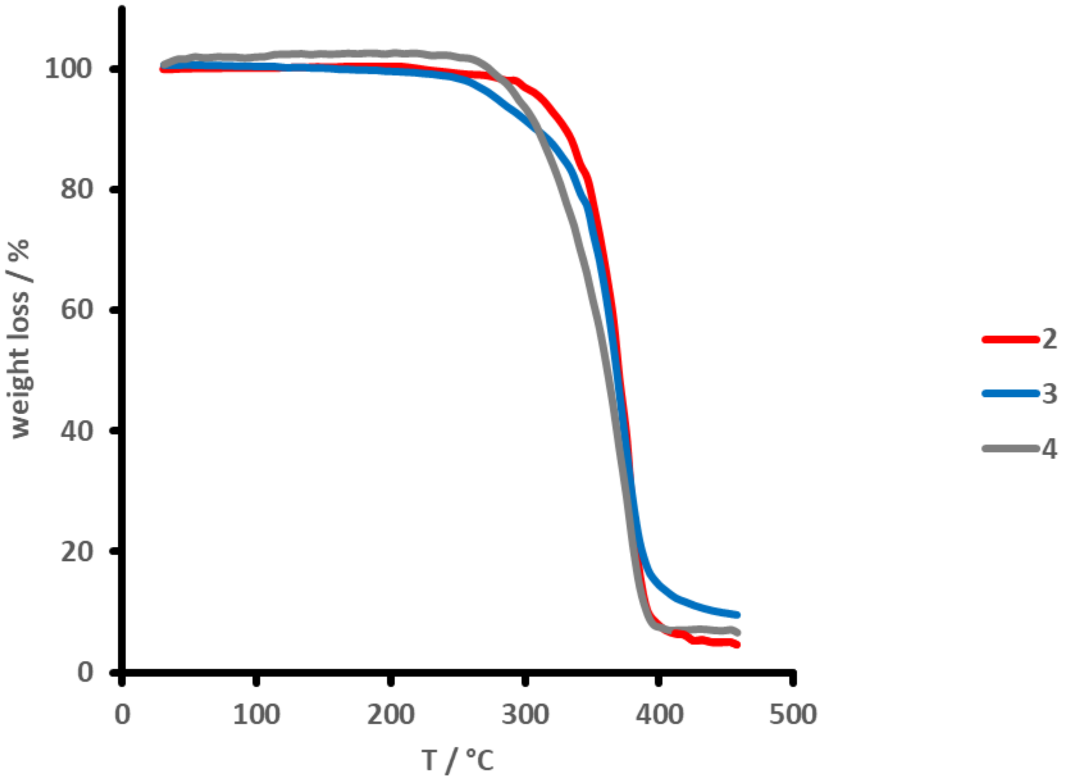

Figure S6. TGA curves for polymers **2** – **4** in N_2 at 10 °C/min.

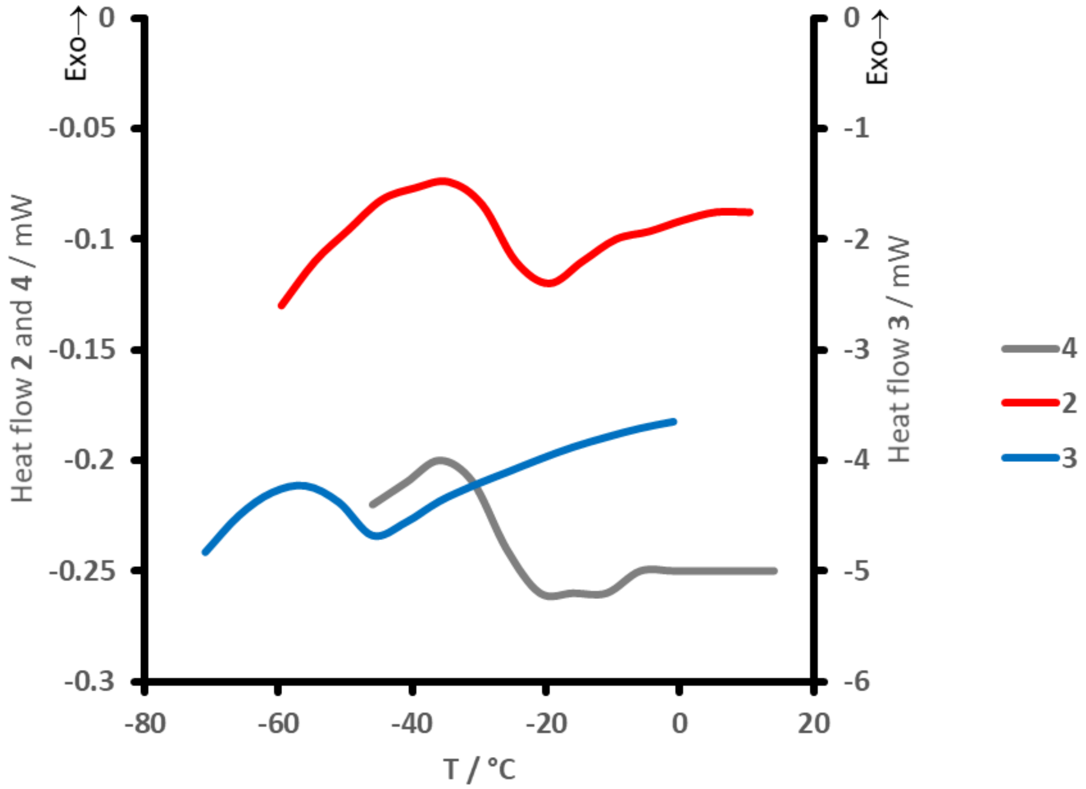

Figure S7. DSC curves for polymers **2** – **4** in N_2 at 10 °C/min; graphs correspond to the respective T_g regions recorded during the measurement cycle.
